# Detecting Emotional Arousal and Aggressive Driving Using Neural Networks: A Pilot Study Involving Young Drivers in Duluth

**DOI:** 10.3390/s24227109

**Published:** 2024-11-05

**Authors:** Md Sakibul Hasan Nahid, Tahrim Zaman Tila, Turuna S. Seecharan

**Affiliations:** Department of Mechanical and Industrial Engineering, University of Minnesota Duluth, Duluth, MN 55812, USA; nahid004@d.umn.edu (M.S.H.N.); tahrim138022@gmail.com (T.Z.T.)

**Keywords:** electrodermal activity, neural networks, aggressive driving, young drivers

## Abstract

Driving is integral to many people’s daily existence, but aggressive driving behavior increases the risk of road traffic collisions. Young drivers are more prone to aggressive driving and danger perception impairments. A driver’s physiological state (e.g., fatigue, anger, or stress) can negatively affect their driving performance. This is especially true for young drivers who have limited driving experience. This research focuses on examining the connection between emotional arousal and aggressive driving behavior in young drivers, using predictive analysis based on electrodermal activity (EDA) data through neural networks. The study involved 20 participants aged 18 to 30, who completed 84 driving sessions. During these sessions, their EDA signals and driving behaviors, including acceleration and braking, were monitored using an Empatica E4 wristband and a telematics device. This study conducted two key analyses using neural networks. The first analysis used a comprehensive set of EDA features to predict emotional arousal, achieving an accuracy of 65%. The second analysis concentrated on predicting aggressive driving behaviors by leveraging the top 10 most significant EDA features identified from the arousal prediction model. Initially, the arousal prediction was performed using the complete set of EDA features, from which feature importance was assessed. The top 10 features with the highest importance were then selected to predict aggressive driving behaviors. Another aggressive driving behavior prediction with a refined set of difference features, representing the changes from baseline EDA values, was also utilized in this analysis to enhance the prediction of aggressive driving events. Despite moderate accuracy, these findings suggest that EDA data, particularly difference features, can be valuable in predicting emotional states and aggressive driving, with future research needed to incorporate additional physiological measures for enhanced predictive performance.

## 1. Introduction

Driving is one of the most important daily life activities. According to the National Highway Traffic Safety Administration (NHTSA), drivers aged 18 to 30 years old are involved in higher (14%) fatal crashes than any other age group. There are around 13.3 million young drivers on the road in the US who are more prone to aggressive driving [1]. Aggressive driving behavior leads to crash risk. Being involved in a car accident is worrisome, and the aftermath of a car crash compounds the situation. People face much hassle, from injury to medical bills, property damage, and lost wages. While, with the implementation of safety legislation (i.e., seat belt usage and drunk driving laws), injurious and fatal road traffic collisions (RTCs) have decreased, these only work to a certain level. As a result, our attention is focused on determining the causative factors related to driving behavior to reduce fatal road crashes.

Aggressive driving behavior is one of the main reasons for crash risk. There can be various reasons for a driver to be involved in aggressive driving. One of the main reasons relates to the driver’s emotions (i.e., fatigue, stress, anger etc.) [2]. Emotions can have a significant impact on driving behavior. Different emotions can influence a driver’s attention, decision-making, risk perception, and overall driving behavior.

## 2. Literature Review

It is well known that human factors, such as distracted driving, impaired driving, and speeding are the main reasons for road traffic accidents. Although in-vehicle technologies exist, recent studies focus on the driver, such as investigating the role of the driver’s personality traits and physiological states on their driving style and, thus, their propensity toward being involved in road traffic accidents.

### 2.1. Personality and Driving Style

An online survey of 439 drivers (ages 18 to 89) examined the perceived road safety risks posed by timid driving behaviors and their links to driver traits (anxiety, anger) and demographics (age, mileage, gender) [3]. Unexpected and overly cautious braking was seen as moderately risky. Older, male, and irritable drivers viewed slow, overly cautious behavior as more hazardous. No significant link was found between driver traits and erratic braking dangers.

Dangerous, anxious, and irate–high-speed styles were positively associated with unsafe driving, while cautious driving was negatively related to hazardous conduct and positively related to good driving [4].

According to the multiple regression analysis, aggressive driving was negatively related to age and positively associated with neuroticism [5]. Age, driving experience, and depression, as well as age, driving experience, and neuroticism, were significantly associated. Simple slope testing indicated that perhaps depression may make older, experienced drivers more aggressive. Neuroticism led to more aggressive driving in those with more experience than those with less.

Hasaninasab et al. investigated the use of psychology in traffic safety to find the effect that personality traits have on the number of accidents [6]. Two hundred college students completed a questionnaire divided into three sections: personal information, questions related to the subject under review, and the Neo test. Using structural equations, the maximum likelihood method, and testing the hypotheses using the AMOS software, the study found significant associations between personality traits and the number of fines and between personality traits and the number of accidents [6].

### 2.2. Driving Style and Emotional Arousal

Driving behavior depends on several parameters: drivers’ mood, focus, road conditions, weather, traffic, etc. Emotional arousal is one of the reasons behind aggressive driving behavior. Poor driving habits are associated with aggressive behavior. To examine the conceptual model of variables and discover the link between aggressive behavior and risky driver conduct, structural equations and the decision tree approach were used to determine the different degrees of dangerous and aggressive driving behavior [7]. Age, attitude, personality traits, hazardous and aggressive driving, and negative emotions and perceptions strongly affected aggressive and dangerous driving behavior. Consequently, the most important variable in the structural equation model, attitude, is associated with aggressive conduct with 84% accuracy for neural networks and unsafe driving with 75% accuracy for SVM.

A recent study used a driving simulator and the Multidimensional Driving Style Inventory (MDSI) to examine the correlation between driving style and biological responses to negative emotions. These findings demonstrated a significant association, with EEG showing the highest correlation. The participants with a nervous and anxious driving style exhibited substantial biological changes, while those with a reckless driving style showed minimal variations [8].

A comprehensive review of fatigue studies involving bus and truck drivers revealed significant findings. The review encompassed various approaches, including biochemical and psychophysiological tests, acoustic speech analysis, continuous heart rate monitoring, and questionnaire studies. These approaches indicated notable psychophysiological, speech, and subjective changes associated with driver fatigue, shedding light on symptoms experienced during prolonged driving as attributable to fatigue [9].

Another study examined the impact of fatigued and drunk driving on drivers’ physical characteristics. It involved 25 participants and measured parameters such as systolic blood pressure, heart rate, eyesight, and reaction times. The results showed that fatigue driving had a greater impact on heart rate and reaction times, while drunk driving affected parameters such as blood pressure, reaction times, and depth perception [10].

Various methods for estimating vehicle acceleration could enhance Advanced Driving Assistance Systems (ADAS) for improved traffic safety. However, many models overlook individual driver characteristics and driving behavior diversity. This study proposes a machine learning model that incorporates driving behavior analysis to improve acceleration estimation accuracy [11].

A study found that higher pre-driving arousal led to better driving behavior, while frequent braking increased post-drive arousal. Sunshine and having passengers boosted well-being. Low pre-driving arousal negatively impacted driving behavior, and monotonous driving situations at high speeds with low cognitive demand increased the risk of tiredness and impaired driving, suggesting the need for timely interventions to enhance well-being while driving [12].

The research found that drivers’ tension increases in specific road segments, including the transition to tunnel entrances, tunnel themselves, and open road portions. The analysis of speed profiles revealed that vehicles often accelerated before exiting a tunnel and slowed down before entering one, with the transition and tunnel segments showing the highest levels of stress [13].

### 2.3. Use of Wearable Devices in Emotional Arousal Detection

Stress represents the brain’s responses to some factors: an individual’s traits and surroundings [14]. Stress can be measured in several ways. The conventional approach for evaluating stress is via cortisol level measurements [15] or via self-reports (e.g., Perceived Stress Scale or PSS) [16]. These approaches cannot be used to quantify stress continually over a lengthy period. In addition, these treatments may involve a visit to a therapist or psychologist [17]. However, recent developments in the detecting capabilities of wearable sensors have been employed by researchers for the continuous monitoring of stress [18,19]. In past research works, several sensors and devices were used to monitor physiological responses; for example, Gjoreski et al. used the E4 Empatica device, which is commercially available. This device was used to detect stress both in the lab and outside the lab [20]; similarly, Mishra et al. used a sensor, Polar H7, with an EDA sensor, to detect stress both in and outside the lab [21,22].

Recent studies indicate a significant increase in using devices that measure physiological arousal via electrodermal activity (EDA) [23]. Although EDA has been recently used to measure emotions in educational contexts, substantial methodological variations exist, revealing inconsistent associations between physiological arousal and learning outcomes, thus indicating the need for explicit guidelines [23]. Detecting stress beforehand can prevent long-term conditions like depression and anxiety [24]. An overview of stress detection techniques based on EDA and machine learning (ML) shows that support vector machines and artificial neural networks achieve high accuracy in predicting stress [24].

Other studies have also used wearable sensors to detect stress rather than determine stress during driving. Under challenging circumstances, student pilots frequently experience stress [25]. As a result, human factors often serve as the primary contributing component in aircraft accidents. In this study, E4 Empatica was used to collect real-time EDA during several flight simulator sessions. According to this study, high demand-related performance correlates with high-stress levels. Additionally, it was seen that students needed help to complete tough jobs despite the fact that they may have performed well overall for the session. The urban stresses discovered using the E4 Empatica in real-world investigations were caused by congested metropolitan locations, risky driving conditions, and traffic jams [3].

A proof-of-concept study continuously monitored electrodermal activity (EDA) and heart rate variability (HRV) to detect stress using a wearable sensor in an ambulatory setting [19]. The participants wore the E4 Empatica for up to 14 days, completing daily surveys on stress, emotions, cravings, pain, and discomfort. The study found that 87.86% of the EDA signals were clean, with strong associations between physiological signals and self-reported outcomes in individuals with alcohol consumption disorder, showing data quality comparable to past research. Recent emotion-recognition studies have utilized wearable sensors. A real-time mobile biofeedback system using the E4 Empatica sensor displayed five basic emotions and provided users with emotional feedback [18]. The iAware system, tested in controlled and uncontrolled conditions, increased emotional self-awareness and decreased prediction error by 3.333% for women and 16.673% for men, highlighting its value in offering real-time biofeedback based on physiological data.

Another study used E4 Empatica, where participants played Tetris at different difficulty levels to induce boredom, tension, and flow. The system was able to detect low-flow vs. high-flow states with 67.50% accuracy and differentiate all the three affective states with a percentage of 49.23%, using end-to-end deep learning architecture [26]. Several other studies have used E4 Empatica for real-time data collection in detecting migraine attacks, infection, health monitoring, emotion recognition, etc. [27,28,29,30].

Studies have been conducted on the impact of emotions on driving behavior [10,31,32]. Emotions can influence driving behavior [8]. However, most of those studies used a driving simulator and questionnaire. Notably, a paper by Najafi et al. investigated the mental attention state of individuals using their EDA, Electrocardiogram (ECG), and Electroencephalogram (EEG) [32]. Our study aimed to collect data in real scenarios while driving on real roads. We investigated the performance of neural networks in predicting emotional arousal using EDA features, aggressive acceleration using EDA features, and aggressive braking using EDA features. Also, along with the questionnaire, accurate emotional arousal data (EDA) have been collected to validate the change in drivers’ emotions.

## 3. Materials and Methods

This study received human research approval from the University of Minnesota’s Institutional Review Board (IRB) with the study code STUDY00013911. Each participant completed a minimum of 5 miles on 5 trips. Most of the driving sessions were held in Duluth, MN. The participants completed a short survey after each driving session. The post-driving session survey is available in the appendix section. Twenty participants (16 male and 4 female) participated in this study. Their age range was between 18 and 30 with a mean of 22.55 and a standard deviation of 1.33. The participants were mostly undergraduate and graduate students at the University of Minnesota Duluth. All the participants were aged between 18 and 30 years. All the participants hold a valid US driving license. The aim was to collect a total of 100 trips (20 participants × 5 trips each).

**Figure 1:**
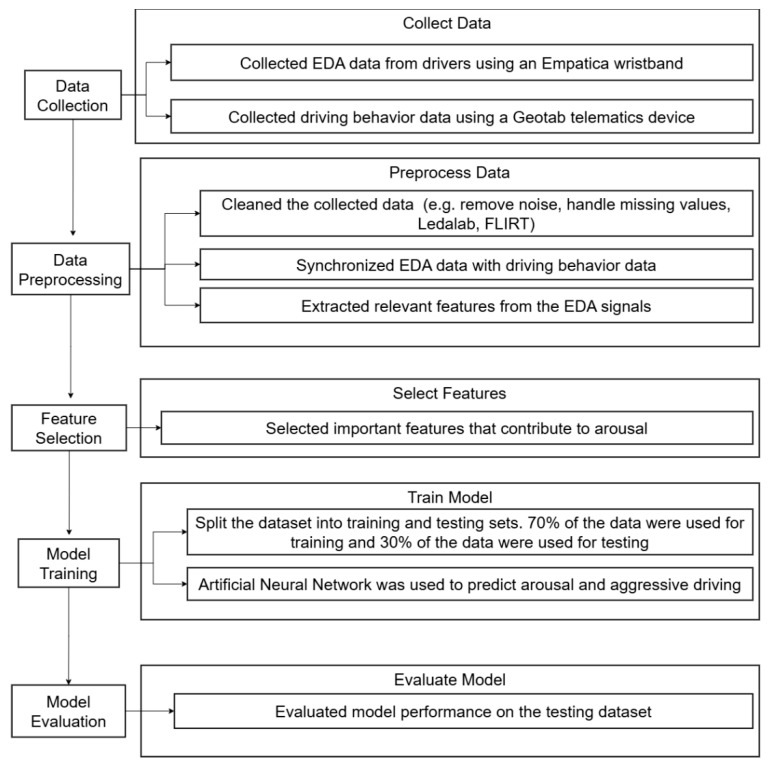
Flowchart of study methodology.

The study methodology is shown in Figure 1.

1. Data collection:
  a. Collect EDA data from drivers;
  b. Collect driving data using a telematics device (e.g., speed, acceleration, braking, and cornering);
2. Label driving behavior as aggressive or non-aggressive;
3. Label emotional arousal state as “aroused” or “not-aroused”.
4. Data preprocessing:
  a. Clean the collected data;
  b. Synchronize EDA data with driving behavior data and survey responses;
  c. Extract relevant features using Ledalab 3.2.5 via MATLAB: R2023b, FLIRT 0.0.3, and Python 3.12.
5. Feature selection:
  a. Select important features that contribute to predicting aggressive driving;
6. Model training:
  a. Split the dataset into training and testing sets;
  b. Train the neural network using the training dataset;
  c. Model evaluation;
7. Evaluate the model performance on the testing dataset using the accuracy, precision, recall, and F1 score.

### 3.1. Equipment—Empatica E4 Wristband

**Figure 2:**
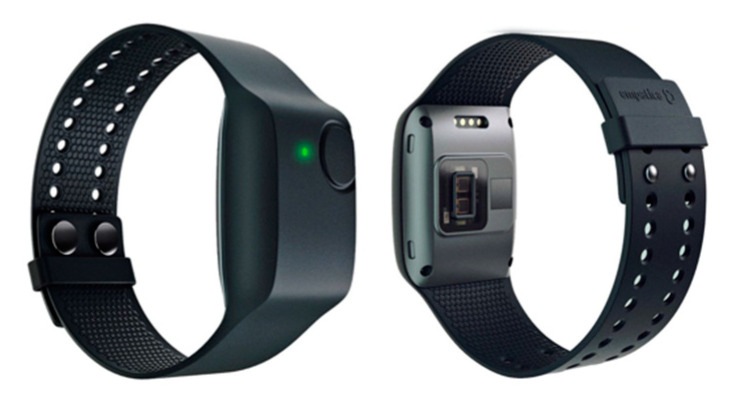
E4 device (Reference: www.empatica.com 27 October 2024).

Two devices were used for data collection: Empatica E4 and a telematics device. See Figure 2. The Empatica E4 is a wristband, high-quality sensor device that falls under the definition of a Class IIa Medical Device, which has low-to-medium risk and is used on the human body for a short time. The Empatica E4 is a device that measures electrical conductance on the skin by applying a small electrical current between two electrodes touching the skin. The E4 wristband is a widely recognized wearable technology that allows for the real-time gathering and analysis of physiological data. The device features various sensors, including a blood volume pulse sensor, an inter-beat interval sensor, and an electrodermal activity sensor, allowing the user to continuously monitor their heartbeat, skin temperature, and electrical skin activity. However, only the electrodermal activity (EDA) measurement taken by Empatica E4 was utilized in creating the dataset. The EDA measurement is expressed in microSiemens (μS). 

### 3.2. Equipment—Telematics Device

**Figure 3:**
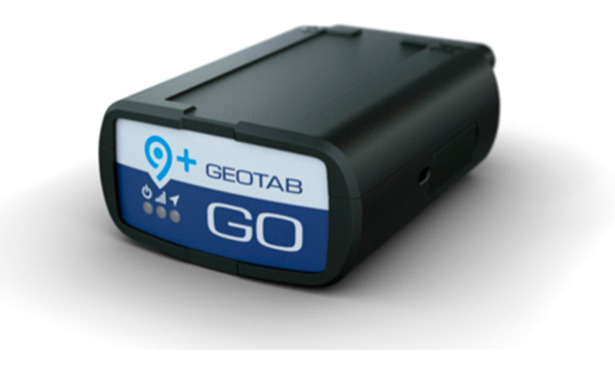
Telematics device (Reference: support.geotab.com 27 October 2024).

For engine data collection, a telematics device was used. See Figure 3. The device was connected to the OBD II port (On-Board Diagnostics, used to obtain engine diagnostics) of the participants’ vehicles. The participants were asked to keep the device installed until all the trips were completed. The telematics device collected several data: acceleration, braking, engine speed, location, fuel level, etc. The data collected by the telematics device were saved on the Geotab website. The data were accessed through a password-protected account. 

### 3.3. Data Collection

**Figure 4:**
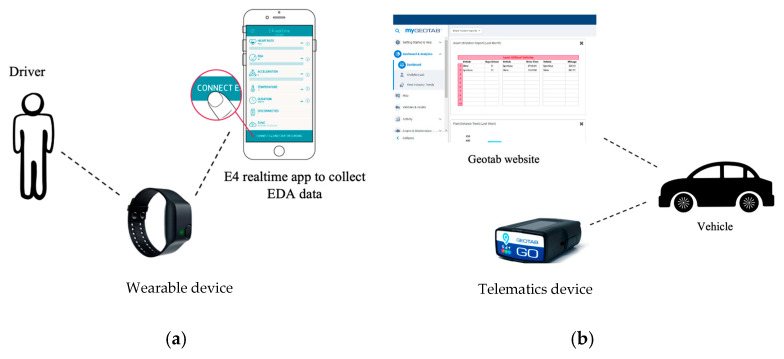
(**a**) EDA data collection setup and (**b**) engine data collection setup.

The participants wore the device on their wrists while driving. The participants were asked to connect to the “E4 realtime” mobile app immediately before starting a driving session. The button on the device was pressed for 3 s to turn it on the device. The device was then connected to a mobile app via Bluetooth. The app was developed by Empatica. After connecting the device to the app, data streaming began, and the participants could start their driving sessions. The data were automatically saved to the password-protected “E4 Connect” website. See Figure 4.

### 3.4. Raw EDA Data vs. EDA Features

Raw EDA data captured the complete physiological responses of the participants and provided a detailed representation of their electrodermal activities. Analyzing raw data allowed for a more comprehensive understanding of the participants’ physiological arousal levels, as it included all the variations and nuances present in the data. It also preserved the temporal characteristics of the physiological responses, such as fluctuations, peaks, and trends. This temporal information can provide insight into the dynamic changes in arousal levels during different driving scenarios or events. It allows for the examination of patterns and trends over time, which can reveal important associations between EDA and driving behavior. Individual differences were accounted for in the electrodermal responses. As each participant may have unique physiological patterns and sensitivities, analyzing raw data enables the exploration of individual variations in the relationship between EDA and driving behavior. This can lead to more personalized and tailored insights. In addition to stress, an individual’s EDA is affected by other environmental factors such as perspiration and caffeine intake. For this reason, EDA features are preferred. EDA features were extracted from the raw EDA signal using the FLIRT 0.0.3 toolkit to separate the signal into its tonic and phasic components. We discuss this feature extraction process in further detail in the section below.

**Figure 5:**
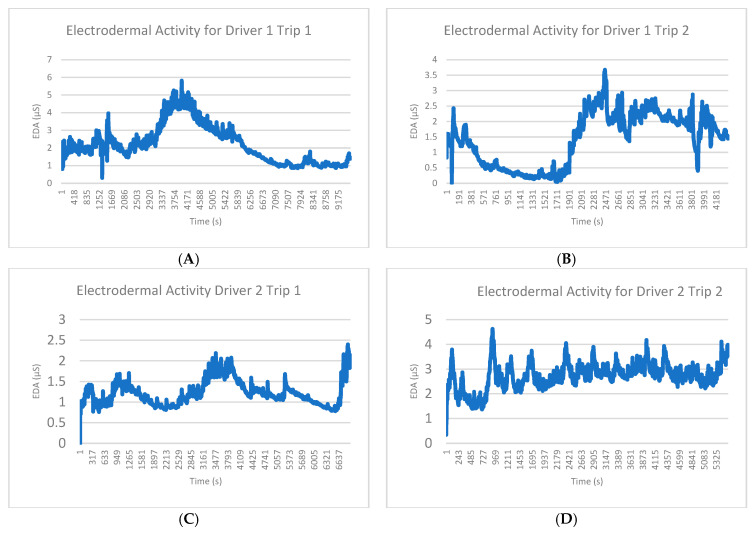
Sample EDA signals for two trips for two drivers. (**A**) Electrodermal activity (EDA) for Driver 1 during Trip 1. EDA values range from approximately 1.0 µS to 6.0 µS, showing significant fluctuations in response to driving events, particularly in the middle of the trip. (**B**) Electrodermal activity (EDA) for Driver 1 during Trip 2. EDA signals exhibit less variability compared to Trip 1, with values fluctuating between 0.5 µS and 3.5 µS. (**C**) Electrodermal activity (EDA) for Driver 2 during Trip 1. EDA values range from 1.0 µS to 2.5 µS. (**D**) Electrodermal activity (EDA) for Driver 2 during Trip 2. The EDA signals for Driver 2 exhibit greater variability during this trip, with values reaching up to 4.0 µS, illustrating the EDA differences in the same subject.

The raw EDA signal from each individual varied. Figure 5 shows sample raw EDA signals from two drivers. The provided graphs (A, B, C, and D) illustrate the variability of EDA signals across trips and subjects. For example, in A, Driver 1’s EDA readings during Trip 1 ranged from approximately 1.0 µS to 6.0 µS, with significant fluctuations around the middle of the trip. Figure 5B shows the EDA readings for Driver 1 during Trip 2, where the fluctuations are smaller, ranging from 0.5 µS to 3.5 µS, demonstrating intra-subject variability between trips. Figure 5C displays the EDA signals for Driver 2 during Trip 1, with values ranging between 1.0 µS and 2.5 µS, while Figure 5D illustrates Driver 2’s EDA during Trip 2, where readings reach up to 4.0 µS, highlighting the variability between different the subjects.

Despite the observed variability, commonalities were evident across the different trips and subjects. In all four figures, distinct peaks in EDA corresponded to high-arousal moments during the trips, indicating a shared physiological response for happy, sad, angry, or stressful driving events. Although the magnitude and baseline values differed across the subjects and trips, these consistent peak patterns suggested a common reaction to driving behaviors.

EDA features, such as tonic mean, tonic max, and phase mean, provide summarized measures that capture specific aspects of electrodermal activities. These features condense the raw data into meaningful and interpretable metrics, simplifying the analysis process. These features offer a standardized representation of the physiological arousal levels across the participants. By using common metrics, comparing, and generalizing, the findings across the different individuals became easier. This standardization facilitated the identification of consistent patterns and trends in the relationship between EDA features and driving behavior.

EDA feature data reduce the dimensionality of a dataset compared to raw EDA data. This simplification can be advantageous for modeling as it reduces computational complexity and helps avoid overfitting. It allows for the more efficient analysis and modeling of the data.

### 3.5. EDA Data Analysis and Feature Extraction

The EDA data were visualized using a MATLAB: R2023b toolkit known as “Ledalab: v3.2.5”, a self-conducted skin conductance analysis software. After completing the driving sessions, the data were downloaded from the PI’s E4 Connect account. First, Ledalab: v3.2.5 was used for signal visualization. An EDA.csv file was imported to obtain an associated signal.

**Figure 6:**
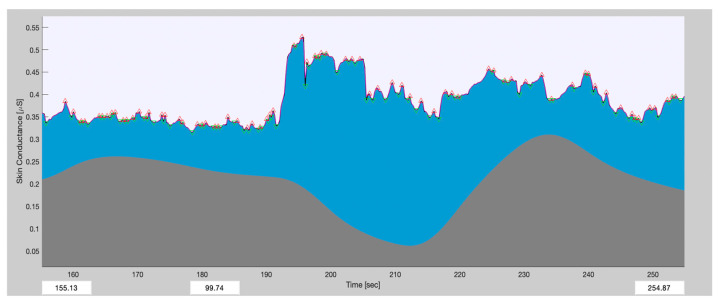
An example of phasic and tonic signals for a driving session. The phasic and tonic signals of the EDA signal are shown: the upper signal (blue section) refers to the phasic signal, and the lower smooth rising signal (gray section) refers to the tonic signal.

Phasic signals were generated when the participant was involved in some abrupt irregular moment (sudden break, sudden disturbance from other vehicles, etc.). Phasic signals are associated with short-term events and occur in the presence of discrete environmental stimuli. The tonic signal would show the smooth rise in aggressive behavior. The progression of the signal was thought to be due to the combination of two distinct factors: a low-frequency background driver that changed very gradually over time (from seconds to minutes) and a rapidly fluctuating phasic component that experienced fluctuations within seconds. The phasic and tonic signals of the above-mentioned signal are shown below: the upper signal (blue section) refers to the phasic signal, and the lower smooth rising signal (gray section) refers to the tonic signal. See Figure 6.

Figure 6 shows a portion of an EDA signal for a driving session. This signal is from the 155th second to the 254th second. From this signal, there appears to be some emotional arousal from the 190th second.

From the signal, the baseline data and the data at the time of arousal were separated manually into multiple datasets. The data were separated with the help of the time span. One EDA signal was the raw signal received when the driver completed one trip. Therefore, that signal’s length depended on the length of the trip. For this work, we focused on the trip lengths of approximately 5 miles in length.

From the visual MATLAB: R2023b signal, the peaks and the flat signal values were separated. If there were multiple peak data files and multiple flat data files, the peak files and the flat files should have been merged into two files: one for all the peak data files, one for all the flat data files.

In the context of extracting EDA features, the FLIRT 0.0.3 toolkit was used as the platform for data analysis and feature extraction [33]. EDA data preprocessing is crucial because the raw data collected by wearables is often prone to noise, missing values, and artifacts. FLIRT 0.0.3 employs various methods to clean and enhance data, ensuring accurate analysis and reliable features for machine learning (ML) models.

FLIRT 0.0.3 is an open-source, free, Python 3.1.2-based toolkit that is specifically designed for processing physiological data, particularly from commercially available wearable sensors. The FLIRT 0.0.3 toolkit employs two Extended Kalman filter techniques, the Extended Kalman filter (EKF) and the particle filter (PF), for removing artifacts and filtering out noise. Then, a modular method combining low-pass filtering and artifact identification techniques was implemented.

The Extended Kalman Filter (EKF) combines the sensor data with a model of the EDA signal to remove noise and artifacts. It uses a state–space model to represent the true physiological signal, including components like skin conductance and sweat diffusion. The Kalman filter reduces noise in a signal by combining real-time measurements with a theoretical model of the signal. The Extended Kalman filter (EKF), a variant of the Kalman filter, assumes that the state and noise variables follow a normal distribution.

The particle filter (PF) is another model-based filtering technique that does not have this requirement, making it more suitable for dealing with signals and situations with highly non-Gaussian noise, particularly in the context of wearable signals. The low-pass filter removes high-frequency noise by retaining only the frequencies below a certain threshold, typically set below 0.5 Hz for EDA signals.

Motion artifacts and irregularities in the data can heavily skew the analysis. FLIRT 0.0.3 offers two built-in methods to automatically detect and correct these artifacts. Within the FLIRT 0.0.3 toolkit, EDAexplorer is a model that detects artifacts based on the morphology of the EDA signal using features such as rise time and rate of change. It labels each segment of data as “artifact”, “questionable”, or “clean”. Within the FLIRT 0.0.3 toolkit, Ideas-Lab UT is a model that uses logistic regression to classify each 5 s window of EDA data as clean or artifact-laden, allowing gaps to be interpolated when necessary. Two elements make up the EDA signal: the skin conductance response (SCR), also referred to as the phasic component, and the skin conductance level (SCL), also known as the tonic component. This toolkit uses cvxEDA, to split EDA data into its phasic and tonic components. The cvxEDA algorithm models an EDA signal as a combination of SCL, SCR, and noise, and solves a convex optimization problem to extract components.

EDA features from both the baseline dataset and aggressive dataset were generated using Python 3.12. Each table that was generated had 1 row and 44 columns containing different EDA features. Following previous work that used EDA features [34], in this work, the EDA features that were used to see the changes between the baseline values and high EDA values are described below:

- Mean phasic—mean value of the phasic component of the EDA signal;
- Mean tonic—mean value of the tonic component of the EDA signal;
- STD phasic—standard deviation of the phasic driver component;
- STD tonic—standard deviation of the tonic driver component;
- Max phasic—maximum value of the phasic driver curve;
- Max tonic—maximum value of the tonic driver curve.

Since each feature had a baseline and high value, twelve feature variables were defined. See Table 1.

An additional six variables were calculated as the difference between the high and baseline values. It is hypothesized that if the driver’s emotional arousal increases, then the difference between the high and baseline values will increase. Therefore, six additional variables were defined. Furthermore, electrodermal behavior can vary significantly across different subjects. One individual’s baseline might be another’s high period. See Table 2.

## 4. Results

A total of 20 drivers (16 male and 4 female) participated in this study between the age range of 16 and 30. Each driver was asked to complete 5 trips, each 5 miles long. Eighty-three trips with complete data (survey responses, EDA signal, and driving data) were recorded. Although 20 drivers signed the consent form to participate in the study, 90 survey responses were received. We then matched the date and time reported on the survey with the date and time of the EDA and trips. Due to errors in recording by the study participants and a couple of EDA signals that were abnormally high, we had to discard some data. In the end, we had complete data for 83 trips. According to the survey, the participants chose their mood while driving. There was a total of five options for mood: stressed, fatigued, angry, happy, and calm. According to our literature review, the emotional states of stress, fatigue, and happiness were identified as emotional arousal and were termed as “arousal”, and the mood “calm” was considered to be “non-arousal”.

The predictions of emotional arousal, aggressive braking, and aggressive acceleration were performed using a neural network with a sigmoid activation function. The sigmoid function, which has an output range of 0 to 1, can be used to identify arousal levels. If the sigmoid’s output exceeded 0.5, the prediction was rated as “arousal”, “aggressive braking”, and “aggressive acceleration” (1); otherwise, it was classified as “non-arousal”, “ non-aggressive braking”, and “non-aggressive acceleration” (0).

### 4.1. Arousal Prediction Using Neural Networks—18 Predictor Variables

**Figure 7:**
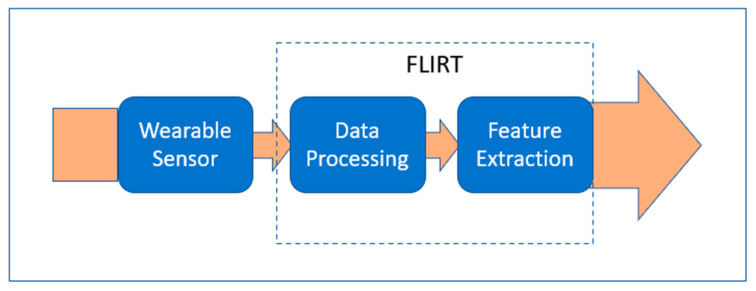
Key functions of FLIRT 0.0.3 tool [33].

**Figure 8:**
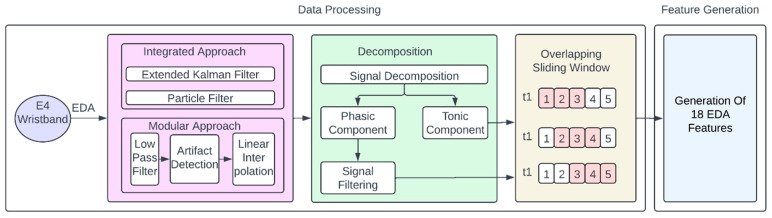
EDA signal processing pipeline.

The 12 EDA feature variables and the 6 difference variables were used as predictor variables to predict arousal (a total of 18 predictor variables). The dataset had these values for 83 trips. The dataset was split into training and testing data. Seventy percent of the data were used to train the Artificial Neural Network (ANN) model and 30% of the data were used to test the model. Using all 18 predictor variables, the ANN model predicted arousal with 65% accuracy. Table 3 shows the results from the model. Figure 7 shows an output from Python 3.12 that shows the weight of each feature in predicting arousal. In the latter sections of this paper, we fitted neural network models using the top 10 features according to Python 3.12 and the 6 difference variables (PhMeanDiff, TMnDiff, PhStdDiff, TnStdDiff, PhMxDiff, and TnMxDiff). See Figure 8.

### 4.2. Arousal Prediction Using 10 Significant Features

**Figure 9:**
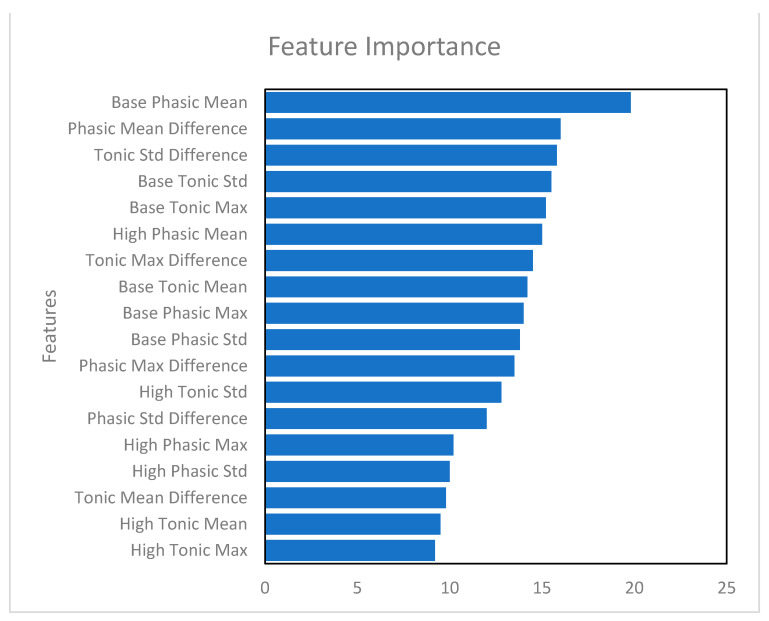
Important features based on their weight to predict arousal.

The first neural network model we fit uses the 10 significant features, as identified in Figure 9, to predict arousal, aggressive acceleration, and aggressive braking. 

**Figure 10:**
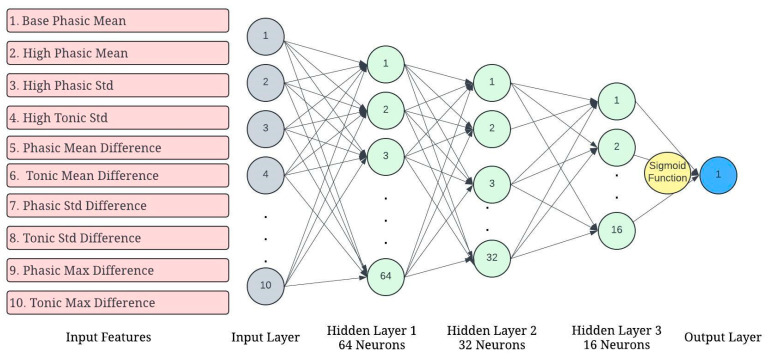
Neural network architecture for predicting arousal, aggressive acceleration, and aggressive braking using 10 physiological features extracted from EDA signals.

We performed a five-fold cross-validation to evaluate the performance of the neural network model and to ensure that overfitting was not an issue. Cross-validation is a well-established method for model validation and helps in assessing how well a model generalizes to unseen data. In particular, five-fold cross-validation was chosen, as it strikes a good balance between bias and variance, while being computationally efficient for our dataset size and model complexity. In machine learning, five-fold cross-validation is a widely accepted practice [35]. It provides a reliable performance estimate while effectively managing computational cost. See Figure 10.

### 4.3. Arousal Prediction with the Six Difference Features Using ANN

**Figure 11:**
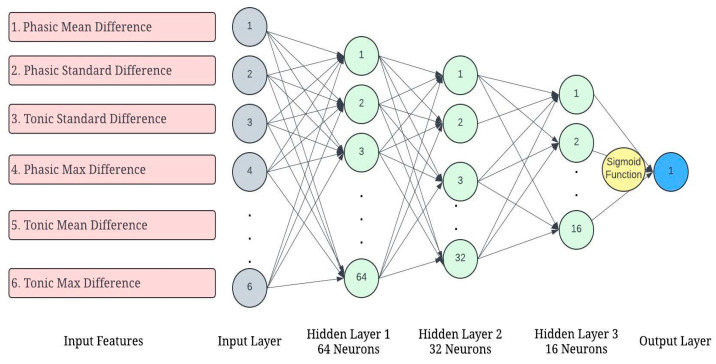
Neural network architecture for predicting arousal, aggressive acceleration, and aggressive braking using the 6 difference features.

The Artificial Neural Network (ANN) model was used to predict arousal levels based on 6 predictor variables for 83 trips: phase mean difference (PhMeanDiff), tonic mean difference (TMnDiff), phasic standard deviation difference (PhStdDiff), tonic standard deviation difference (TnStdDiff), phasic maximum difference (PhMxDiff), and tonic maximum difference. These variables describe the differences between high and baseline values in both phasic and tonic metrics, which are important for understanding physiological changes related to arousal. These variables were then used to predict aggressive acceleration and aggressive braking. See Figure 11.

Comparing the outputs from Table 4 and Table 5, the neural network using the 6 “difference variables” had a higher average precision than the model using the 10 significant features. Precision is simply interpreted as a measure of quality and recall as a measure of quantity. A higher precision means that 75% of the predictions were correct. 

### 4.4. Aggressive Acceleration Prediction Using the Significant EDA Features

The telematics device collected the acceleration and braking data of the vehicles during the driving sessions. The median acceleration value for each driver for each trip was recorded and compared to the median acceleration value of all the accelerations recorded from all the drivers for all the driving sessions. If a driver’s median acceleration for a trip was greater than the overall median, this was labeled as “aggressive acceleration”. Similarly, if a driver’s median braking for a trip was greater than their overall median, this was labeled as “aggressive braking”. The top 10 features from Figure 9 were used to predict aggressive acceleration and aggressive braking. With the best 10 features, the model predicted aggressive braking with 65% accuracy. However, the precision, recall, and F1 scores were low at 0.40. See Table 6 and Table 7.

### 4.5. Aggressive Acceleration and Braking Prediction with Six Difference Variables

Again, ANN was used to predict aggressive acceleration and aggressive braking using the six “difference” variables as features. The model showed the same result as before in terms of accuracy. Compared to the model using the 10 significant features, the reduced model with 6 predictor variables had a higher performance in terms of precision. Looking at the average F1 scores, using the 10 features was better at predicting aggressive acceleration. See Table 8 and Table 9.

## 5. Discussion

### 5.1. Summary and Contribution of the Work

This work presents a unique application of using EDA data to predict aggressive acceleration and braking. In particular, this study evaluates the effectiveness of neural networks in predicting emotional arousal and aggressive driving behaviors, specifically acceleration and braking, using electrodermal activity (EDA) data collected from young drivers. We attempted to reduce the number of predictor variables (EDA features) by focusing only on the significant features, as reported by Python 3.12, and the six “difference variables” that compare a driver’s high and baseline EDA values. These findings not only offer insight into the relationship between physiological responses and driving behaviors but also provide practical implications for improving driving performance, contributing to the broader understanding of how emotional states impact driving performance. A future application of this work might be to monitor a driver’s emotional state before driving and build systems that might assist in reducing a driver’s stress to reduce the occurrence of aggressive driving, which may ultimately reduce their chance of being involved in a dangerous accident.

Twenty participants (16 male and 4 female) took part in this study between the age range of 18 and 30, with a mean of 22.55 and an SD of 1.33. A total of 84 trips were conducted by the participants. Each participant conducted 5 trips of approximately 5 miles in length. Out of 100 trips, 17 trips were disregarded due to missing data. After each trip, the participants completed a survey about their mood before, during, and after driving. There were five options for mood: stressed, fatigued, angry, happy, and calm. Stressed, fatigued, angry, and happy moods were deemed to be emotional arousal and were termed as “arousal”, and the mood “calm” was considered to be “non-arousal”.

The ANN analysis involved using two sets of predictor variables: (1) 10 significant EDA features and (2) 6 difference features that represented the change between high and baseline EDA values. The results were assessed using key performance metrics, such as accuracy, F1 score, recall, and precision. A five-fold cross-validation was used with the neural network model to predict emotional arousal, aggressive acceleration, and aggressive braking. In general, when predicting emotional arousal, the neural network using the 6 “difference” variables had a higher precision than the model using the 10 significant features. When predicting aggressive acceleration and aggressive braking, the model using the six variables had a higher precision.

When employing the 10 significant EDA features, the model predicted the test cases with an average accuracy of 63%; an average precision of 41%, and an average recall of 66%. The average F1 score was 51%. The 66% average recall indicates that the model performed better than guessing at identifying positive cases—in this case, emotional arousal. However, the lower average precision reduced the F1 score. On the other hand, the model’s performance with the six difference features was similar, with an F1 score of 0.5, precision of 0.75, and a recall of 0.42. With the 10 significant features, this model performed better at identifying emotional arousal than the model using the 6 difference features.

For aggressive acceleration, the model utilizing the 10 significant EDA features yielded a moderate F1 score of 0.58, with precision at 0.47 and recall at 0.75. The high recall of 0.75 means 75% of the actual aggressive acceleration events were identified. The precision of 0.47 means 47% of the positive predictions were correct. On the other hand, the model leveraging six difference features had an average accuracy of 0.60, an average precision of 0.65, an average recall of 0.37, and an average F1 score of 0.47. The lower F1 score indicates that the model was not better than random guessing.

For aggressive braking, both the models demonstrated limited performance. When utilizing the 10 significant EDA features, the model achieved an F1 score of 0.47, with both precision and recall at 0.38 and 0.71, respectively. The model using six difference features performed similarly, with a slight increase in precision to 0.41 and an increase in recall to 0.66, with an F1 score of 0.51. The recall values in both the models suggest that both the models were able to identify the true aggressive braking events.

The comparison between the models using all the EDA features and those using the six difference features reveals some interesting insights. While both models performed similarly in terms of overall accuracy, the models based on the six difference features generally showed better precision, particularly in predicting aggressive acceleration. This suggests that these difference features, which capture the change in physiological arousal relative to a baseline, might be more sensitive to the dynamics of driving behaviors linked to emotional states.

However, the trade-off between precision and recall observed in these models highlights the challenges of using EDA data alone for such predictions. Interestingly, the lower precision in models using the 10 significant EDA features, which indicates a higher rate of false positives, might be advantageous in this context. Detecting non-aggressive drivers as aggressive (false positives) is preferable to missing actual aggressive behaviors (false negatives), as it allows for preemptive measures that could enhance road safety. This approach ensures that potential risks are addressed, albeit at the cost of some inaccuracies, rather than failing to detect genuine aggressive driving incidents that could lead to accidents.

These findings highlight the potential of using EDA data, particularly the difference features, to predict emotional arousal and certain aggressive driving behaviors. However, the moderate accuracy, and variable precision and recall scores, suggest that relying solely on EDA may not lead to highly reliable predictions. The combination of EDA with other physiological data such as HRV to improve model performance will be explored in future research.

### 5.2. Limitations and Future Work

The present study had a few limitations. It had only 20 participants, constituting a small number of drivers that limited the generalization of findings across the population. Additionally, there was an imbalance between the male and female drivers. We focused our recruitment efforts on Mechanical and Industrial Engineering students, which has a larger proportion of male students. We continue to recruit participants to increase our database.

This research used only one physiological measure of emotional arousal: electrodermal activity (EDA). Even though EDA is a useful measure for estimating activity within the sympathetic nervous system, the full scope of both emotional and cognitive processes that influence driving behavior were not necessarily covered. The developed neural network models showed only moderate performance (60% accuracy), and further improvements can be expected after the addition of other physiological parameters such as heart rate variability (HRV).

We attempted to reduce the number of EDA features to 10 significant features and then to 6 “difference features”. However, it might be useful to keep all 16 features and then use a standard dimensionality reduction technique like Principal Component Analysis to reduce the number of predictor variables. Additionally, we maintained one architecture of the neural network. Increased accuracy might be possible by testing different architectures.

Future research should address these limitations by increasing the sample size to enhance the applicability of these findings and enable more detailed analysis. Furthermore, incorporating additional physiological measures, such as HRV, could offer a more comprehensive understanding of drivers’ emotional and cognitive states, potentially leading to more precise and reliable predictive models. We tested the neural network using ten significant features and then the difference variables. Future research will use principal component analysis to reduce the dimensionality of the EDA signal.

## Figures and Tables

**Table 1:** The twelve feature variables.

Variable	Variable Name	Description
BPhMean	Base phase mean	Mean of the phasic signal during the baseline period
HPhMean	High phase mean	Phasic mean during the high period
BTnMean	Base tonic mean	Tonic mean during the baseline period
HTnMean	High tonic mean	Tonic mean during the high period
BPhStd	Base phasic std	Phasic standard deviation during the baseline period
HPhStd	High phasic std	Phasic standard deviation during the high period
BTnStd	Base tonic std	Tonic standard deviation during the baseline period
HTnStd	High tonic std	Tonic standard deviation during the high period
BPhMx	Base phasic Max	Phasic maximum during the baseline period
HPhMx	High phasic Max	Phasic maximum during the high period
BTnMx	Base tonic max	Tonic maximum during the baseline period
HTnMax	High tonic max	Tonic maximum during the high period

**Table 2:** Six “difference variables”.

Variable	Variable Name	Description
PhMeanDiff	Phase Mean Difference	Difference between high and baseline phasic means
TMnDiff	Tonic Mean Difference	Difference between high and baseline tonic means
PhStdDiff	Phasic Std Difference	Difference between high and baseline phasic standard deviations
TnStdDiff	Tonic Std Difference	Difference between high and baseline tonic standard deviations
PhMxDiff	Phasic Max Difference	Difference between high and baseline phasic maximums
TnMxDiff	Tonic Max Difference	Difference between high and baseline tonic maximums

**Table 3:** Performance of the ANN model to predict arousal.

Model	Accuracy	Precision	Recall	F1
ANN	0.65	0.73	0.73	0.73

**Table 4:** Performance of the ANN model to predict **arousal** with **10 significant features**.

Model	Accuracy	Precision	Recall	F1
ANN	0.63 (+/−0.1)	0.41 (+/−0.1)	0.66 (+/−0.08)	0.51 (+/−0.08)

**Table 5:** Performance of the ANN model to predict arousal with the 6 “difference variables”.

Model	Accuracy	Precision	Recall	F1
ANN	0.61 (±0.09)	0.75 (±0.2)	0.42 (±0.1)	0.5 (±0.15)

**Table 6:** Accuracy, precision, recall, and F1 scores when ANN is used to **predict aggressive acceleration** using the **10 significant features**.

Model	Accuracy	Precision	Recall	F1 Score
ANN	0.60 (±0.12)	0.47 (±0.06)	0.75 (±0.2)	0.58 (±0.12)

**Table 7:** Accuracy, precision, recall, and F1 score results using ANN to **predict aggressive braking** with **10 significant features**.

Model	Accuracy	Precision	Recall	F1 Score
ANN	0.60 (±0.13)	0.38 (±0.08)	0.71 (±0.32)	0.47 (±0.15)

**Table 8:** Accuracy, precision, recall, and F1 score using ANN and **6 “difference variables”** to predict **aggressive acceleration**.

Model	Accuracy	Precision	Recall	F1 Score
ANN	0.60 (±0.11)	0.65 (±0.08)	0.37 (±0.1)	0.47 (±0.11)

**Table 9:** Accuracy, precision, recall, and F1 score using ANN and **6 “difference variables”** to predict **aggressive braking**.

Model	Accuracy	Precision	Recall	F1 Score
ANN	0.63 (±0.10)	0.41 (±0.08)	0.66 (±0.08)	0.51 (±0.08)

## Data Availability

The original data presented in this study are openly available in Seecharan, Turuna; Tila, Tahrim Zaman. (2023). EDA Driving Data and Survey Responses. Retrieved from the Data Repository for the University of Minnesota (DRUM), https://doi.org/10.13020/9dre-8p52, accessed on 27 October 2024.
